# The impact of medically tailored meals and nutrition therapy on biometric and dietary outcomes among food-insecure patients with congestive heart failure: a matched cohort study

**DOI:** 10.1186/s40795-022-00602-y

**Published:** 2022-10-03

**Authors:** Lauren Belak, Caroline Owens, Margaret Smith, Eric Calloway, Laura Samnadda, Heartley Egwuogu, Stacie Schmidt

**Affiliations:** 1 Department of Medicine, Massachusetts General Hospital, 55 Fruit Street, Boston, MA 02114 USA; 2 Department of Anthropology, Emory University, 1557 Dickey Dr, Atlanta, GA 30322 USA; 3 Department of Medicine, University of California San Diego, 9500 Gilman Dr. La Jolla, San Diego, CA 92093 USA; 4 Gretchen Swanson Center for Nutrition, 14301 FNB Pkwy Suite 100, Omaha, NE 68154 USA; 5 Open Hand Atlanta, 181 Armour Dr NE, Atlanta, GA 30324 USA; 6 Grady Memorial Hospital, 80 Jesse Hill Jr Dr SE, Atlanta, GA 30303 USA

**Keywords:** Congestive heart failure, Medically tailored meals, Nutrition intervention, Food insecurity, Program evaluation

## Abstract

**Background:**

To evaluate the impact of home-delivered, medically tailored meals and medical nutrition therapy among food-insecure patients following hospitalization for congestive heart failure by comparing clinical outcomes to a retrospectively matched cohort.

**Methods:**

Patients at high risk for readmission and food insecurity received up to three months of medically tailored meals and medical nutrition therapy after discharge. Pre-intervention and post-intervention weight, body mass index, blood pressure, and dietary intake were assessed. A combination of difference-in-difference and logistic regression models were used to compare changes between cohorts and evaluate impact attributable to the program.

**Results:**

Thirty-nine program participants were compared to a matched cohort of 117 unexposed patients. Participants experienced a marginal reduction in body mass index and an increase in systolic and diastolic blood pressure; however, these results were not statistically significant. To determine relevance to clinical cut-offs, logistic regressions were used, demonstrating that exposure to the intervention resulted in higher odds of a categorical reduction in blood pressure (OR: 1.85), though this did not reach statistical significance (95% CI: 0.67–5.32). Pre vs. post trends indicated that more-healthful foods and drinks increased numerically or remained similar to baseline, while less-healthful foods decreased numerically or remained similar to baseline.

**Conclusions and implications:**

These findings highlight the need for more longitudinal research on medically tailored meals and medical nutrition therapy interventions using clinical outcomes while setting realistic suggestions for program implementation. This study additionally illustrates the promise of integrating electronic medical record data and matched cohorts into medical nutrition program evaluation within the health sector.

**Supplementary Information:**

The online version contains supplementary material available at 10.1186/s40795-022-00602-y.

## Background

As the United States population ages, the prevalence of congestive heart failure (CHF) continues to rise [1]. According to the National Health and Nutrition Examination Survey (NHANES) administered from 2013 to 2016, an estimated 6.2 million American adults live with CHF, which is notably greater than the estimated 5.7 million adults from NHANES data three years prior [2]. Trends suggest that the prevalence of CHF will increase by 46% from 2012 to 2030, leaving more than 8 million Americans with this condition and costing the United States (US) nearly 69.8 billion a year [3].

The rising prevalence of CHF has been matched by increasing rates of hospitalization. Nearly 25% of patients hospitalized with CHF are readmitted within one month of discharge, [4, 5], with readmission rates rising over time [4]. Recurrent hospitalizations are linked to increased mortality rates [6], lower patient satisfaction [7], and exorbitant expense, costing Medicare up to $17 billion per year in hospital bills [8]. With 30-day mortality rates increasing over the past several years [9], approaches to improve outcomes among patients recently hospitalized for CHF are needed.

Importantly, dietary behaviors have been identified as preventable causes of rehospitalizations among patients with CHF [10]. Sodium restriction, for example, has been a hallmark of clinical practice guidelines for cardiovascular risk reduction for years [11]. Yet, in an effort to reduce consumption of sodium-containing foods, it is hypothesized that patients with CHF may unintentionally exacerbate micronutrient deficiencies [12], which in turn leads to poorer health outcomes [13]. Now prevalent in more than 15% of those admitted for CHF [10], malnourishment is associated with poorer quality of life, increased hospitalization duration, rate of rehospitalization, and long-term mortality [10, 13].

The Dietary Approaches to Stop Hypertension (DASH) diet, which is high in fruits and vegetables, whole grains, fish and poultry, and low in saturated fats and sodium, has long been implemented in the management CHF [9, 14], and may help prevent the micronutrient deficiencies prevalent in this population. Compliance with this diet is not only associated with a lower risk of developing heart failure [14], but also decreased blood pressure [15] and lower cardiovascular and metabolic risk [16, 17]. Despite its prominent health benefits, DASH uptake has been poor across the nation [18, 19], likely due to greater expense and more limited availability of DASH-accordant foods relative to their more calorie-dense counterparts [18, 20]. DASH diet compliance is further limited by socioeconomic status, with NHANES data revealing lowest accordance scores among disadvantaged groups [21].

A further barrier to consumption of heart healthy diets linked to socioeconomic status and malnourishment is food insecurity. Severity of food insecurity ranges across a spectrum of limited access, to insufficient quantity and/or quality of food, to anxiety about having enough food to eat [22]. Food insecurity is a notable public health problem, as an estimated 10.5% of US households in 2019 did not have access to enough food to support a healthy lifestyle [23]. Recent estimates suggest that food insecurity prevalence across the US has increased during the COVID-19 pandemic [24], further magnifying inequities in cardiovascular and metabolic diseases [25]. Unhealthy eating patterns and dependence on nutritionally inadequate foods can significantly compromise dietary quality [22, 26], and subsequently predispose food insecure patients to cardiovascular disease (CVD) [27]. Illustrating this inequity, a recent Feeding America report found that food-insecure seniors were 57% more likely to have CHF than their food-secure counterparts [28]. Given that adherence to a healthy diet is essential for heart failure management, food insecure patients with CHF are at heightened risk for poor disease control and costly, preventable healthcare utilization.

An innovative and upcoming strategy to address both food insecurity and barriers to a balanced diet among those with diet-related chronic disease is the provision of medically tailored meals (MTM). MTM delivery consists of home-delivered meals crafted by Registered Dietitian Nutritionists (RDN) that align with the nutritional needs of one’s medical condition [29]. Participation in a MTM program has been associated with significantly fewer hospitalizations and skilled nursing admissions among vulnerable patients, as well as decreased overall medical expenditure [29]. In one study, chronically ill, Medicare and Medicaid beneficiaries who received 6 months of MTM had 50% fewer inpatient admissions and 70% fewer emergency department visits than those not enrolled in the program, with an average net savings of more than $200 per patient [30]. Furthermore, a recent trial among food insecure patients with type II diabetes found that MTM significantly improved dietary quality, food security, and frequency of self-reported hypoglycemic episodes [31]. Despite the success of MTM among those with other chronic diseases, little is known about its effect among food-insecure individuals with CHF, who remain a particularly under-supported and understudied population.

To our knowledge, no interventions to date have addressed whether home-delivered meals following discharge may improve nutritional status and clinical outcomes among food-insecure patients with CHF. Hummel and colleagues conducted the first and only known pilot study (GOURMET-HF) evaluating the efficacy of 4 weeks of home-delivered, sodium-restricted meals among patients discharged from a CHF hospitalization [10]. Their intervention was found to be safe, feasible and efficacious, demonstrating directionally favorable benefits in heart failure symptoms, mobility, and 30-day readmissions [8]. However, nutritional support was offered for only one month following discharge, and was not paired with nutrition education, which has been shown to improve knowledge, self-efficacy, and behavioral changes among patients [32]. The impact of several months of nutritional support coupled with nutritional education among food-insecure patients with CHF warrants further investigation.

To address this gap, our safety-net hospital partnered with a community-based organization to provide three months of home-delivered MTM and adjunctive Medical Nutrition Therapy (MNT) to recently hospitalized, food-insecure patients with CHF who were at high risk for readmission. The primary purpose of this quality improvement initiative was to enhance clinical care by helping patients conform to evidence-based dietary recommendations for CHF. As the first pilot study of its kind, we secondarily sought to evaluate the feasibility, safety, and degree of patient engagement in this novel approach to care for CHF. We hypothesized that among those receiving MTM and MNT, intake of foods high in saturated fat and sodium would decrease, fruit and vegetable consumption would increase, and readmission rates would be improved relative to national average Medicare CHF readmission rates. Additionally, we hypothesized that receipt of MTM and MNT and associated dietary improvements would promote physical health outcomes, leading to reductions in weight, BMI, and blood pressure among program participants compared to a matched cohort.

## Methods

### Intervention

Grady Memorial Hospital partnered with Open Hand Atlanta, a social service organization that offers prepared meal delivery and nutrition education programs to citizens of Atlanta with diet-related disease. From May 2019 to July 2020, Open Hand prepared, packaged, and home delivered MTM, defined as meals designed by a RDN that reflect evidence-based dietary guidelines for the management of CHF [33]. In addition to MTM, patients received MNT with a RDN once per month for three months following hospitalization for CHF.

Those who completed the intervention in full received 3 home-delivered MTM per day for a total of 3 months. Fresh meals were delivered once per week, or patients could request fresh-frozen options for convenience or storage purposes. MTM consisted primarily of whole grains, fresh fruits and vegetables, fish and poultry, with limited sodium and saturated-fat containing foods. Sodium content was restricted to less than 660 mg per meal and 2,000 mg per day in accordance with American Heart Association (AHA) guidelines [34]. Daily intake was approximately 1600 cal excluding beverages and snacks. Because some participants had comorbid pre-diabetes or Type 2 diabetes mellitus, all meals also followed the American Diabetes Association guidelines for consistent carbohydrate intake and balanced protein and fat macronutrients [35].

Patients also received MNT, consisting of three individual sessions with a RDN at baseline and following 6 and 12 weeks of their participation. During these sessions, RDNs educated patients on heart healthy eating behaviors, assessed nutritional status and body weight, and promoted patients’ adherence to evidence-based dietary guidelines for CHF.

### Analytical sample

#### Program participants

**Fig. 1:**
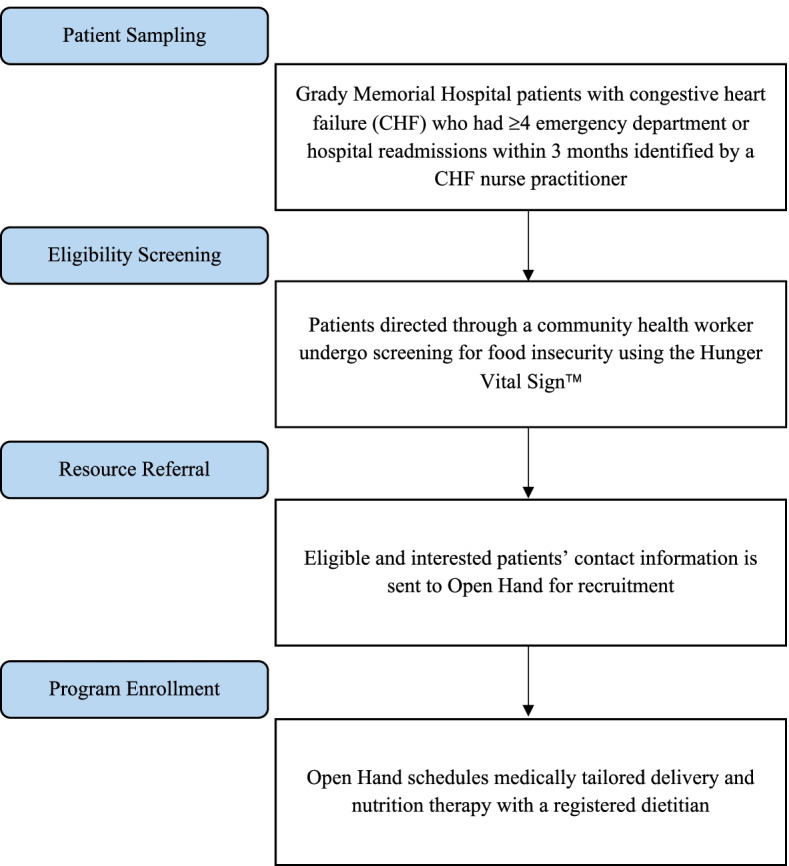
Sampling and enrollment procedures for the Medically Tailored Meal and Medical Nutrition Therapy pilot intervention

Patients were recruited from Grady Memorial Hospital, Atlanta’s largest safety-net healthcare system that provides care to low-income and uninsured populations who experience food insecurity at higher rates than those in the greater metro-area [36]. Patients prospectively eligible for the program were currently hospitalized for CHF, had demonstrated ≥ 4 readmissions or Emergency Department (ED) visits within the past 3 months, and were food-insecure (Fig. 1). Patients who met the readmission criteria were prospectively identified by a CHF nurse practitioner during their hospitalization. A community health worker on the CHF clinical team then screened those individuals for food insecurity by the two-question Hunger Vital Sign™ validated ​​screening tool [37] in person or via telephone within 30 days of discharge. Eligible and interested patients’ referral information was then sent to Open Hand Atlanta for enrollment. Client services staff at Open Hand Atlanta then scheduled delivery of medically tailored meals and MNT with a staff dietitian for those who enrolled. Sessions with the dietitian took place at Grady Memorial Hospital. Those who were experiencing homelessness were excluded from participation because meals could not be delivered to the same residence consistently.

#### Non-exposed matched cohort

To provide a more robust analytical sample and a comparison group, this study used an intervention-recipient matched cohort design in which individuals who received MTM and MNT were matched with patients with CHF who were identified through EMR data and did not receive the intervention. Matched patients were retrospectively identified based on biologic sex, age (± 3 years), Community Needs Index (CNI) score (a composite measure of five zip-code based socioeconomic indicators associated with health disparities), and number of all-cause inpatient admissions (± 2 admissions) in the previous year. For each patient exposed to the intervention, three patients that matched these criteria were identified. To preserve patient privacy, a de-identified analytic data set was created. Therefore, the final analytic sample included all individuals exposed to the intervention for any amount of time (*n* = 39) and matched patients (*n* = 117). This study was deemed exempt from review by the Institutional Review Board at Emory University as part of a quality improvement initiative and form of program evaluation. All participants consented for participation in the intervention verbally, as this research involved no more than minimal risk.

### Measures

#### Biometric data

Electronic medical record (EMR) data were used to provide biometric and relevant demographic information recorded as part of a clinic or hospital visit during the intervention period for all intervention participants and matched controls. In addition to the demographic criteria outlined in the matching schema, race and ethnicity were included as covariates in the analyses to account for potential impacts of structural racism and discrimination on health outcomes [38, 39]. Body mass index was derived from height (converted to meters) and weight (in kilograms) using the standard BMI formula with these measurements: weight [kg] / height [m]^2^. From this calculation, BMI categories were created using cut-offs defined by the Centers for Disease Control. Blood pressure categories were generated following the guidelines of the American Heart Association for both pre-and post-intervention time points. Notably, biometric outcome measures were not available for all individuals, resulting in missing values that constrained sample sizes in the regressions. Missingness for biometric outcomes ranged from 13 intervention patients and 48 matched controls to 17 intervention patients and 68 matched controls; missingness and its impact on sample demographics are detailed in Supplemental Tables 1 and 2.

#### Pre-post dietary changes

To assess daily intake frequencies of foods and beverages, patients who participated in the intervention were asked to complete the FRESH Foods Survey [40], a validated dietary assessment, to indicate the number of times they consumed 23 different individual foods over the past week at baseline and upon completion of the program. This survey is not under license and is freely available to the public [40]. The primary outcomes assessed were patients’ pre-post changes in dietary intake of individual foods and food groups, measured in times per day. To derive food group scores, daily intake frequencies of individual food were summed to produce food groups with similar nutritional values following similar procedures used in past research [40].

### Statistical methods

#### Biometric data

Descriptive statistics, including means, frequencies, and cross-tabulations, were used to assess all individuals’ demographic and biometric characteristics at both pre- and post-timepoints. Difference-in-difference models were used to analyze the effect attributable to intervention exposure by comparing the difference in differences with change in biometrics modeled continuously. The difference in difference approach is commonly used in natural experiments and intervention settings as a means of estimating a causal effect of interest (in this case, the impact of the nutrition intervention on health outcomes) [41]. In these models, causal effects are estimated based on the difference in average differences between the two comparison groups. In this case, the difference in difference compares the average pre-post difference of program participants to the matched patient cohort. The causal effect is estimated as the difference-in-differences, represented in the regression coefficient on the interaction term in model summaries. The primary independent variable in each biometric difference-in-difference model was exposure to the nutrition intervention for any amount of time, such that participants were considered “cases” (equal to 1) and the matched individuals were considered “controls” (equal to 0). For these models, the dependent or outcome variables of interest were body mass index, systolic blood pressure, and diastolic blood pressure.

As aforementioned, all biometrics were measured and collected by clinicians and modeled continuously for the difference-in-difference analyses. The difference-in-difference for body mass index is defined ​​as follows:

For individual *i* in hospital *j* in cohort *c* (where cohorts represent exposure to the intervention):

$${\varvec{Y}}{\varvec{i}}{\varvec{j}}{\varvec{c}}={\varvec{X}}{\varvec{i}}{\varvec{\beta}}+{\varvec{P}}{\varvec{O}}{\varvec{S}}{\varvec{T}}{\varvec{c}}+\boldsymbol{ }{\varvec{\delta}}\boldsymbol{ }\left({\varvec{I}}{\varvec{N}}{\varvec{T}}{\varvec{E}}{\varvec{R}}{\varvec{V}}{\varvec{E}}{\varvec{N}}{\varvec{T}}{\varvec{I}}{\varvec{O}}{\varvec{N}}{\varvec{j}}\boldsymbol{ }\boldsymbol{*}{\varvec{P}}{\varvec{O}}{\varvec{S}}{\varvec{T}}{\varvec{c}}\boldsymbol{ }\right)+\boldsymbol{\alpha }{\varvec{j}}+\boldsymbol{ }{\varvec{\epsilon}}{\varvec{i}}{\varvec{j}}$$

Defined as such, Yijc refers to the biometric outcome of interest. X*i* refers to participant covariates outlined above. INTERVENTION*j* represents intervention exposure, while POSTc indicates the time-point at which the measure was taken (pre-or post-intervention). The interaction term (INTERVENTIONj * POSTc) captures the causal effect of interest (i.e., the effect of exposure to the nutrition intervention on health outcomes of interest).

Additionally, logistic regressions were used to assess changes in individuals’ BMI and blood pressure categories before and after the intervention. This approach was chosen given that categorization of health outcomes may provide a more clinically relevant or meaningful representation of change. More specifically, logistic regressions modeled odds of categorical improvement in blood pressure such that any reduction from hypertensive or elevated levels pre-intervention to a lower category post-intervention was counted as an improvement. All models were adjusted for age, sex, inpatient admissions, and CNI scores to account for residual imbalance after matching and missingness of data. Our analyses followed the intention-to-treat principle whereby individuals who enrolled in the intervention were analyzed as part of the intervention even if participation ended before completion of the full three months of MTM and MNT. Model estimates are presented with 95% confidence intervals (CI). Statistical analyses were performed using R software (R Core Team, 2020). Power estimates suggest that our sample size is sufficiently powered to detect a medium effect (Cohen’s d of 0.5).

#### Pre-post dietary changes

Pre-post changes in dietary variables were assessed using the Wilcoxon signed rank test and by calculating Cohen’s d effect sizes. As expected for an exploratory analysis of a small sample (*n* = 11), there were few changes statistically significant at the 0.05 alpha level. Changes that were less than an alpha level of 0.10 and |> 0.500| effect size were therefore deemed meaningful changes. All data analysis was completed with SAS software (Cary, NC). With a sample size of 11, the probability that the Wilcoxon tests would detect an effect at an alpha level of 0.10 is lower than the conventional 0.8 threshold, suggesting our tests are underpowered.

## Results

The analytic sample comprised 39 program participants and 117 matched patients; however, the missing biometric data from the EMRs substantially impacted the sample size for several models. In addition, supplementary analyses were performed among 23 program participants who received meals for the full three months of the program and attended at least one MNT session. The sample size and respective sociodemographic information associated with each key biometric outcome are noted in Supplemental Tables 1 and 2. The majority of program participants and matched controls were non-Hispanic Black (94.9%), male (64.1% and 63.2%, respectively), and had an average age of approximately 64 years, with a range of 37 to 92 years old (Table 1). In the previous year, individuals had an average of approximately 3 inpatient hospital admissions. During the intervention period, 8 individuals (20.5%) exposed to the intervention were hospitalized for cardiovascular-related events, which is lower than the most recent 90 day nation-wide CHF readmission rate of 34.6% [4]. Fourteen of the matched individuals were hospitalized (12.0%). Among those hospitalized for cardiovascular-related events, the average number of hospitalizations was not significantly different using an independent samples t-test [95% CI (-0.46—1.35)] with an average of 1.88 and 1.43 hospitalizations for the intervention and matched groups respectively.

**Table 1:** Matched demographic characteristics of the medically tailored meals and medical nutrition therapy pilot cohort (*n* = 156)

	**Intervention Cohort** **(*N* = 39)**	**Matched Control Cohort** **(*N* = 117)**	**Overall** **(*N* = 156)**
**Age**			
Mean (SD)	63.8 (12.6)	64.2 (12.4)	64.1 (12.4)
Median [Min, Max]	63.0 [37.0, 91.0]	63.0 [37.0, 92.0]	63.0 [37.0, 92.0]
**Sex**			
Female	14 (35.9%)	43 (36.8%)	57 (36.5%)
Male	25 (64.1%)	74 (63.2%)	99 (63.5%)
**Race and Ethnicity**			
Black or African American, Non-Hispanic	37 (94.9%)	111 (94.9%)	148 (94.9%)
White or Caucasian, Non-Hispanic	2 (5.1%)	4 (3.4%)	6 (3.8%)
Hispanic	0 (0%)	2 (1.7%)	2 (1.3%)
**Community Needs Index Score**			
4	20 (51.3%)	54 (46.2%)	74 (47.4%)
5	19 (48.7%)	60 (51.3%)	79 (50.6%)
Missing	0 (0%)	3 (2.6%)	3 (1.9%)
**Inpatient Admissions in the Previous Year**			
Mean (SD)	3.30 (2.36)	2.67 (2.47)	2.82 (2.45)
Median [Min, Max]	3.00 [0, 8.00]	2.00 [0, 9.00]	2.00 [0, 9.00]
Missing	2 (5.1%)	0 (0%)	2 (1.3%)

Due to the reliance upon retrospectively-pulled EMR data, the time of biometric measurement relative to the intervention start and stop points varied substantially across individuals (Table 2). Weight, and therefore BMI, data were available on average 42 days prior to intervention start date for all individuals included in the analyses (Table 2). Longer average time-lapses occurred for blood pressure, with an average of 52 days between measurement and intervention start dates. The average time between intervention stop and post-measurement was longer, with 76 days for weight and BMI and 79 days for blood pressure measurements, respectively.

**Table 2:** Days between intervention time-points and clinical measurement of biometric outcomes (*n* = 156)

	**Days Between Outcome Pre-Measurement and Intervention Start Date**	**Days Between Intervention Stop Date and Outcome Post-Measurement**
**BMI**		
Mean (SD)	41.5 (44.9)	75.7 (49.9)
Median [Min, Max]	23.0 [0, 179.0]	72.0 [1.0, 180.0]
Missing	56 (35.8%)	56 (35.8%)
**Blood Pressure**		
Mean (SD)	52.3 (53.0)	78.6 (50.2)
Median [Min, Max]	69.0 [0, 182.0]	80.0 [0, 176.0]
Missing	75 (48.7%)	75 (48.7%)

### Body mass index and weight change

**Fig. 2:**
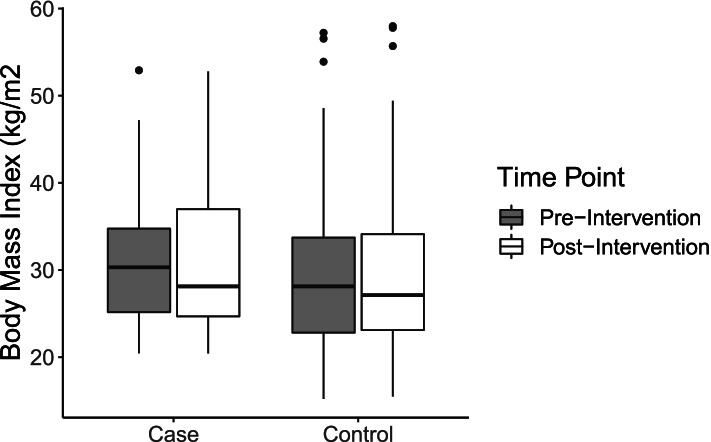
Distribution of body mass index among cases and controls at pre- and post-intervention timepoints

The difference between the effects of pre-and post-intervention differences and being exposed to the intervention estimates the effect of interest. On average, individuals exposed to the intervention had higher BMI at both pre-intervention and post-intervention timepoints compared to their matched peers (Table 3; Fig. 2). After controlling for age, sex, race, ethnicity, CNI scores, and inpatient admissions in the previous year, individuals exposed to the intervention did not have significantly different BMI scores than their matched peers (31.5 kg/m^2^ [9.4 kg/m^2^] vs. 29.5 kg/m^2^ [9.9 kg/m^2^]) (Table 3). The difference-in-difference coefficient suggests that the true effect of the intervention was a 0.04 kg/m^2^ reduction in body mass index, though this effect was not statistically significant (Table 4). To assess any clinical or practical significance in these differences, discerning threshold change in weight was also relevant. Logistic regressions were used to estimate odds of a 2.5% reduction in body weight comparing the pre-and post-weight measures. We chose the 2.5% cut-off based on previous intervention studies using a 5% metric over a six-month intervention period [42]. Controlling for age and sex, being exposed to the intervention was associated with greater, though not significant, odds of losing 2.5% of body weight (Table 5- OR: 1.18, 95% CI: 0.44–3.07).

**Table 3:** Average biometric measures pre-and post-intervention stratified by intervention exposure

	**Intervention Cohort** **(*N* = 39)**		**Matched Control Cohort** **(*N* = 117)**	
	**Pre**	**Post**	**Pre**	**Post**
**Biometric Outcomes**				
**BMI**				
Mean (SD)	31.8 (8.4)	31.5 (9.4)	29.8 (9.6)	29.5 (9.9)
Median [Min, Max]	30.3 [20.4, 52.9]	28.1 [20.4, 52.8]	28.1 [15.2, 57.2]	27.1 [15.5, 58.0]
Missing	13 (33.3%)		46 (41.0%)	
**SBP**				
Mean (SD)	126 (23.1)	125 (25.4)	136 (24.1)	133 (20.4)
Median [Min, Max]	127 [85.0, 168]	122 [86.0, 175]	134 [90.0, 215]	131 [95.0, 196]
Missing	17 (43.6%)		68 (58.1%)	
**DBP**				
Mean (SD)	72.1 (12.3)	75.2 (15.6)	79.5 (15.4)	75.5 (12.4)
Median [Min, Max]	71.5 [51.0, 105]	70.5 [47.0, 111]	81.0 [48.0, 118]	77.0 [47.0, 98.0]
Missing	17 (43.6%)		68 (58.1%)	

*BMI* Body mass index (kg/m2), *SBP* Systolic blood pressure (mmHg), *DBP* Diastolic blood pressure (mmHg)

**Table 4:** Adjusted difference in difference model for BMI

	**Body Mass Index Outcome**		
*Predictors*	*Estimates*	*CI*	*p*
(Intercept)	47.53	40.28 – 54.77	** < 0.001**
Intervention Exposure	1.16	-3.00 – 5.33	0.583
Pre-Post Difference	-0.27	-3.25 – 2.71	0.858
Age	-0.21	-0.32 – -0.10	** < 0.001**
Male	-4.10	-7.00 – -1.20	**0.006**
Race- Hispanic	-6.36	-19.23 – 6.52	0.331
Race- White	-1.41	-10.41 – 7.59	0.757
Community Needs Index 5	-0.36	-3.16 – 2.43	0.798
Community Needs index Missing	-6.16	-18.90 – 6.58	0.341
Inpatient Admissions	-0.48	-1.04 – 0.07	0.086
Difference in Difference	-0.04	-5.90 – 5.81	0.988
Observations	186		
R^2^ / R^2^ adjusted	0.170 / 0.123

**Table 5:** Adjusted odds of losing at least 2.5% of body weight

	**2.5% Loss in Body Weight**		
*Predictors*	*Odds Ratios*	*CI*	*p*
(Intercept)	0.04	0.00 – 0.41	**0.010**
Intervention Exposure	1.18	0.44 – 3.07	0.735
Age	1.04	1.00 – 1.08	**0.038**
Male	1.64	0.66 – 4.29	0.299
Observations	95		
R^2^ Tjur	0.050		

### Blood pressure

**Fig. 3:**
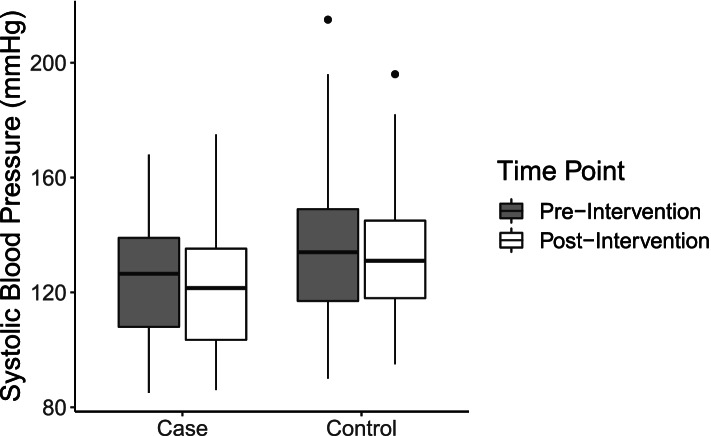
Distribution of systolic blood pressure among cases and controls at pre- and post-intervention timepoints

**Fig. 4:**
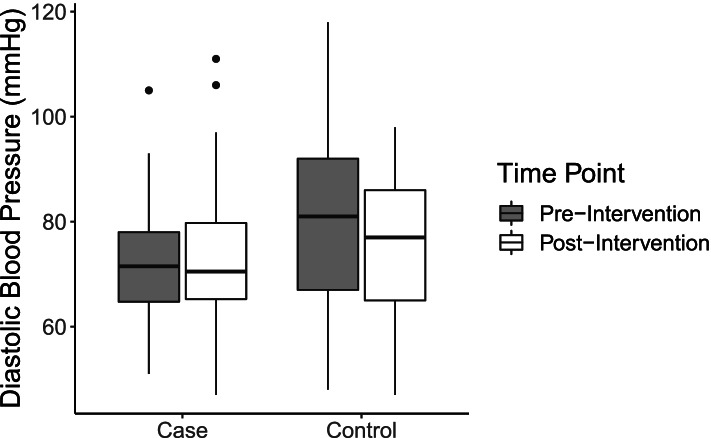
Distribution of diastolic blood pressure among cases and controls at pre- and post-intervention timepoints

Compared to matched controls, individuals exposed to the intervention had lower systolic and diastolic blood pressures, on average, at both timepoints (Table 3; Figs. 3 and 4). In the difference-in-difference models, blood pressure was stratified into continuous systolic and diastolic measurements for all individuals. For both systolic and diastolic outcomes, the difference-in-difference effects suggest that the intervention exposure increased outcome measures, but neither effect was statistically significant (Tables 6 and 7; Figs. 3 and 4). To assess clinically significant change, logistic regressions for odds of categorical blood pressure change were used. Once again, controlling for age and sex, exposure to the intervention resulted in higher odds of categorical reduction in blood pressure (OR: 1.85), though this effect did not reach statistical significance (95% CI: 0.67–5.32) (Table 8).

**Table 6:** Adjusted difference in difference model for systolic blood pressure

	**Systolic Blood Pressure Outcomes**		
*Predictors*	*Estimates*	*CI*	*p*
(Intercept)	121.71	97.86 – 145.55	** < 0.001**
Intervention Exposure	-9.69	-26.23 – 6.84	0.248
Pre-Post Difference	-3.69	-13.00 – 5.61	0.433
Age	0.23	-0.07 – 0.53	0.127
Male	-1.99	-10.64 – 6.65	0.649
Race- White	-23.62	-57.58 – 10.34	0.171
Community Needs Index- 5	0.59	-7.85 – 9.02	0.891
Community Needs Index- Missing	-5.30	-39.18 – 28.58	0.757
Inpatient Admissions	0.20	-1.63 – 2.03	0.828
Difference in Difference	1.59	-15.68 – 18.87	0.855
Observations	138		
R^2^ / R^2^ adjusted	0.076 / 0.003

**Table 7:** Adjusted difference in difference model for diastolic blood pressure

	**Diastolic Blood Pressure Outcomes**		
*Predictors*	*Estimates*	*CI*	*p*
(Intercept)	87.55	75.04 – 100.06	** < 0.001**
Intervention Exposure	-7.08	-14.27 – 0.12	0.054
Pre-Post Difference	-3.94	-9.39 – 1.51	0.155
Age	-0.20	-0.38 – -0.02	**0.026**
Male	4.29	-0.78 – 9.35	0.096
Race- White	-12.84	-32.42 – 6.75	0.197
Community Needs Index- 5	3.24	-1.69 – 8.17	0.196
Community Needs Index- Missing	13.69	-6.16 – 33.53	0.175
Inpatient Admissions	0.06	-1.01 – 1.13	0.910
Difference in Difference	6.09	-4.03 – 16.21	0.236
Observations	138		
R^2^ / R^2^ adjusted	0.131 / 0.070

**Table 8:** Adjusted odds of categorical improvement in blood pressure

	**Categorical Improvement in Blood Pressure**		
*Predictors*	*Odds Ratios*	*CI*	*p*
(Intercept)	2.57	0.21 – 33.13	0.459
Intervention Exposure	1.85	0.67 – 5.32	0.244
Age	0.98	0.95 – 1.02	0.394
Male	0.74	0.27 – 1.98	0.544
Observations	71		
R^2^ Tjur	0.030		

### Dietary changes pre-post intervention

Using our benchmark of meaningful changes (< 0.10 alpha level and |> 0.500| Cohen’s d), participants who completed the standardized food questionnaire pre and post intervention (*n* = 11) showed a statistically significant increase in fruit and milk consumption of 0.71 (*p* = 0.031, Cohen’s d 0.7105) and 0.66 (*p* = 0.016, Cohen’s d 0.7059) times per day, respectively (Table 9). There was also a statistically significant decrease in chips consumption by 0.19 times per day (*p* = 0.063, Cohen’s d -0.8338) (Table 9). None of the changes in other specific foods reached statistical significance.

**Table 9:** Pre-post changes in dietary intake of individual items, measured in times per day (*n* = 11)

Food Group	Timepoint	Mean	Std Dev	Median	Min	Max	N	Difference	S-Statistic	*P*-Value	Cohen's d
**Water**	**Pre**	2.43	0.75	3.00	0.71	3.00	11	0.00	0.00	1.000	0.0000
	**Post**	2.43	0.75	3.00	0.71	3.00					
**Milk**	**Pre**	0.36	0.67	0.00	0.00	2.00	11	0.66	14.00	**0.016**	0.7059
	**Post**	1.03	1.14	0.71	0.00	3.00					
**Milk Alternatives**	**Pre**	0.12	0.31	0.00	0.00	1.00	11	-0.03	-0.05	1.000	-0.0869
	**Post**	0.09	0.30	0.00	0.00	1.00					
**Fruit (F)**	**Pre**	0.53	0.78	0.29	0.00	2.00	11	0.71	10.50	**0.031**	0.7105
	**Post**	1.25	1.18	0.71	0.00	3.00					
**Salad**	**Pre**	0.16	0.15	0.29	0.00	0.29	11	0.06	2.50	0.750	0.3469
	**Post**	0.22	0.22	0.29	0.00	0.71					
**Fried Potatoes**	**Pre**	0.08	0.14	0.00	0.00	0.29	11	0.03	1.50	1.000	0.1870
	**Post**	0.11	0.15	0.00	0.00	0.29					
**Non-Fried Potatoes**	**Pre**	0.18	0.15	0.29	0.00	0.29	11	-0.03	-1.50	1.000	-0.1771
	**Post**	0.16	0.15	0.29	0.00	0.29					
**Vegetables (V)**	**Pre**	0.96	1.17	0.29	0.00	3.00	11	-0.01	0.50	1.000	-0.0111
	**Post**	0.95	0.95	0.29	0.29	3.00					
**Beans**	**Pre**	0.13	0.15	0.00	0.00	0.29	11	0.18	5.50	0.250	0.7563
	**Post**	0.31	0.30	0.29	0.00	1.00					
**Pizza**	**Pre**	0.08	0.14	0.00	0.00	0.29	11	0.03	1.00	1.000	0.1870
	**Post**	0.11	0.15	0.00	0.00	0.29					
**Tacos & Burritos**	**Pre**	0.03	0.09	0.00	0.00	0.29	11	0.00	0.00	1.000	0.0000
	**Post**	0.03	0.09	0.00	0.00	0.29					
**Heat-and-Serve**	**Pre**	0.29	0.59	0.00	0.00	2.00	11	-0.21	-3.00	0.250	-0.4899
	**Post**	0.08	0.14	0.00	0.00	0.29					
**Processed Meat**	**Pre**	0.42	0.59	0.29	0.00	2.00	11	-0.08	-0.50	0.938	-0.1580
	**Post**	0.34	0.35	0.29	0.00	1.00					
**Hamburgers**	**Pre**	0.13	0.15	0.00	0.00	0.29	11	0.00	0.00	1.000	0.0000
	**Post**	0.13	0.15	0.00	0.00	0.29					
**Fried Chicken**	**Pre**	0.16	0.15	0.29	0.00	0.29	11	-0.03	-1.50	1.000	-0.1741
	**Post**	0.13	0.15	0.00	0.00	0.29					
**Whole Grain Bread**	**Pre**	0.38	0.58	0.29	0.00	2.00	11	-0.23	-6.50	0.344	-0.5325
	**Post**	0.14	0.23	0.00	0.00	0.71					
**Cooked Whole Grains**	**Pre**	0.30	0.36	0.29	0.00	1.00	11	-0.16	-5.50	0.313	-0.5173
	**Post**	0.14	0.23	0.00	0.00	0.71					
**Candy & Chocolates**	**Pre**	0.59	0.98	0.29	0.00	3.00	11	-0.17	-0.50	1.000	-0.1818
	**Post**	0.42	0.89	0.00	0.00	3.00					
**Frozen Dessert**	**Pre**	0.14	0.31	0.00	0.00	1.00	11	0.27	2.00	0.625	0.4021
	**Post**	0.42	0.91	0.00	0.00	3.00					
**Cookies & Cakes**	**Pre**	0.28	0.27	0.29	0.00	1.00	11	-0.08	-2.00	0.625	-0.2740
	**Post**	0.20	0.30	0.00	0.00	1.00					
**Chips**	**Pre**	0.30	0.29	0.29	0.00	0.71	11	-0.19	-7.50	**0.063**	-0.8338
	**Post**	0.11	0.15	0.00	0.00	0.29					
**Sugary Cereals**	**Pre**	0.05	0.12	0.00	0.00	0.29	11	0.08	3.00	0.250	0.5839
	**Post**	0.13	0.15	0.00	0.00	0.29					
**Non-Sugary Cereal**	**Pre**	0.03	0.09	0.00	0.00	0.29	11	0.00	0.00	1.000	0.0000

None of the greater food groups (Supplemental Table 3) achieved an alpha level of < 0.10; however, several achieved a Cohen’s d of |> 0.500| including an increased intake frequency of whole fruits and vegetables and whole fruits and vegetables without potatoes, and a decreased intake frequency of high sodium foods and whole grain foods. With the exception of whole grain foods and frozen desserts, general trends indicated that the more-healthful foods and drinks increased numerically or remained similar to baseline, while the less-healthful foods decreased numerically or remained similar to baseline (Supplemental Table 3).

## Discussion

Heart failure is a common cause of hospitalization, and is associated with considerable morbidity and mortality [43]. As readmission rates continue to rise [4], developing novel strategies to address modifiable risk factors are of utmost importance in public health. Despite the known association between poor dietary habits and food insecurity with increased risk of cardiovascular disease [22], few have investigated whether nutritional support following hospital admission may affect dietary behaviors and clinical outcomes among food-insecure patients with CHF. By using a comparison group for evaluation, this study was able to more sensitively control for external factors and unmeasured confounders that may influence change in clinical outcomes over time.

The results of this pilot study shed light on the potential impacts of a novel dietary intervention among this particularly vulnerable and under-supported patient population. Our analyses of this MTM and MNT pilot program did not find statistically significant change in weight, BMI, or blood pressure compared to a matched cohort. However, the improvements in our patients’ dietary intake, along with a 90-day readmission rate less than the national average, suggests that MTM and MNT may be beneficial for clinical status and readmission rates among patients at our local hospital who have been recently hospitalized with CHF. To our knowledge, only one other study has assessed the impact of MTM among patients with CHF. The results of this pilot study reinforce those of the GOURMET-HF trial, illuminating that home delivery of MTM is feasible and low risk for patients with CHF, as 82% (*n* = 32) of those enrolled participated in at least all three months of MTM. Our initiative builds upon this prior research by demonstrating the potential positive effects of a longer duration of nutritional support paired with formal nutrition education following discharge for food insecure patients with CHF.

In exploratory analyses of biometric and anthropometric outcomes among MTM participants across “on-meal” and “off-meal” periods, Berkowitz and colleagues did not find any significant changes during a study with a similar duration to the present study [31]. However, participants in their study self-reported lower food insecurity, fewer days where mental health interfered with quality of life, and improvements in health eating index scores during their “on-meal” periods as compared to “off-meal periods” [29]. Collectively, our findings suggest that significant biometric changes may not be expected in relatively short-term MTM interventions. The demonstrated improvements in dietary quality evident in our subsample and previous research may lead to more substantial and significant improvements in biometric and anthropometric outcomes over a longer intervention or study period. Longitudinal research on the clinical outcomes that may accompany improvements in dietary quality and food insecurity associated with MTMs is needed to better understand these dynamics.

The results of this pilot study should be interpreted in light of several limitations. First, this quality improvement initiative was designed to improve Grady Hospital’s post-discharge care for recently hospitalized patients with CHF, a clinically and nutritionally at-risk cohort of patients from a safety-net hospital. Accordingly, our results may not be generalizable to other healthcare settings or to the greater population of patients with this illness. Others are encouraged to draw their own conclusions about the applicability of our results to their respective institutions. Second, our study relied on EMR data, which contributed to temporal variability in biometric measurement relative to the pre- and post-intervention time-points. Third, the modest program sample size, and the variability in duration of intervention exposure among the program limit generalizability. Fourth, the short time-frame of study limits our ability to assess whether significant changes in biomarkers or anthropometric measurements occur over time or with longer intervention periods. Next, although patients from the matched cohort did not receive the MTM and MNT intervention, these individuals may have received other nutrition interventions such as the Supplemental Nutrition Assistance Program, Meals on Wheels, or food bank assistance. Participant consumption of meals, a critical assumption of MTM programs, was also not assessed during this study. Finally, the present study was not able to assess effects associated with specific program components (for instance, the medically tailored preparation of food, the Medical Nutrition Therapy, or social connection associated with delivery).

While MTM delivery attempts to address food insecurity, unmeasured compounding issues such as poverty, lack of transportation, and lack of adequate housing may persist among many recipients [44]. Therefore, it is conceivable that MTM delivery may have relatively minimal impact on health outcomes when not partnered with more holistic socioeconomic intervention. Previous qualitative research found that MTM participants expressed many of these barriers, suggesting that assistance with other financial barriers beyond food was an important area for contemporaneous intervention efforts [44]. Further qualitative research that explores drivers of health-care utilization and disease management may prove particularly informative for developing interventions tailored to specific drivers.

These limitations are balanced by several strengths. These include the enrollment of a predominantly minority and socioeconomically vulnerable sample of individuals frequently left out of research on nutrition interventions for chronic disease. Overall, this study population was representative of the safety-net hospital population from which it was drawn. This study is also one of few to assess healthcare utilization, dietary quality, and biometric and anthropometric outcomes in conjunction. Finally, the retrospective cohort design of the study provided additional control for unmeasured, confounding factors.

Future research is needed among a larger cohort of patients to determine the true significance of the clinical and/or dietary changes observed, as well as the long-term impact of this intervention. Food insecurity, perceived quality of life, physical activity, and metabolic markers should also be assessed throughout the intervention and during follow up to elucidate its effects more clearly.

## Conclusions

In conclusion, the results of the study have implications for the employment of MTM and MNT for food-insecure patients with CHF at Grady Hospital, and possibly beyond. This intervention may help address the significant barriers to heart-healthy diets encountered by this patient population, such as cost, access, and knowledge of appropriate foods, and help promote dietary compliance. While previous research on MTMs has documented lower health-care costs and utilization among recipients [30, 31], additional research is needed to elucidate the full impact of MTM and MNT on clinical outcomes, particularly among food insecure communities across the nation. Future longitudinal program evaluations with comparison groups may also have important clinical and policy implications, strengthening support for prescribing and reimbursing this novel treatment approach across healthcare and insurance sectors.

## Supplementary Information

**Additional file 1:** **SupplementalTable 1.** Demographics for individuals with pre- and post- weight and BMI data.

**Additional file 2:** **SupplementalTable 2.** Demographics for individuals with pre- and post- blood pressure data.

**Additional file 3:** **Supplemental Table 3.** Pre-Post Changes in DietaryIntake of *Food Groups*, Measured inTimes Per Day (*n*=11).

## Data Availability

The data that support the findings of this study are available from the corresponding author on reasonable request.

## Abbreviations

NHANES: National health and nutrition examination survey
CHF: Congestive heart failure
DASH: Dietary approaches to stop hypertension
CVD: Cardiovascular disease
MTM: Medically Tailored Meals
RDN: Registered dietician nutritionist
MNT: Medical nutrition therapy
BMI: Body mass index
AHA: American heart association
ED: Emergency department
CNI: Community needs index
EMR: Electronic medical record

## References

[1] Tsao CW, Aday AW, Almarzooq ZI, Alonso A, Beaton AZ, Bittencourt MS (2022). Heart disease and stroke statistics-2022 update: a report from the american heart association. Circ.

[2] NHANES - about the national health and nutrition examination survey. 2020. Available from: https://www.cdc.gov/nchs/nhanes/about_nhanes.htm. Cited 2022 Apr 23

[3] Heidenreich PA, Albert NM, Allen LA, Bluemke DA, Butler J, Fonarow GC (2013). Forecasting the impact of heart failure in the United States. Circ Heart Fail.

[4] Khan MS, Sreenivasan J, Lateef N, Abougergi MS, Greene SJ, Ahmad T (2021). Trends in 30- and 90-day readmission rates for heart failure. Circ Heart Fail.

[5] Bernheim SM, Grady JN, Lin Z, Wang Y, Wang Y, Savage SV (2010). National patterns of risk-standardized mortality and readmission for acute myocardial infarction and heart failure. Update on publicly reported outcomes measures based on the 2010 release. Circ Cardiovasc Qual Outcomes.

[6] Setoguchi S, Stevenson LW, Schneeweiss S (2007). Repeated hospitalizations predict mortality in the community population with heart failure. Am Heart J.

[7] Boulding W, Glickman SW, Manary MP, Schulman KA, Staelin R (2011). Relationship between patient satisfaction with inpatient care and hospital readmission within 30 days. Am J Manag Care.

[8] Jencks SF, Williams MV, Coleman EA (2009). Rehospitalizations among patients in the Medicare fee-for-service program. N Engl J Med.

[9] Fonarow GC (2008). Factors identified as precipitating hospital admissions for heart failure and clinical outcomes findings from optimize-hf. Arch Intern Med.

[10] Hummel SL, Karmally W, Gillespie BW, Helmke S, Teruya S, Wells J (2018). Home-delivered meals postdischarge from heart failure hospitalization. Circ Heart Fail.

[11] Eckel RH, Jakicic JM, Ard JD, de Jesus JM, Houston Miller N, Hubbard VS (2014). 2013 aha/acc guideline on lifestyle management to reduce cardiovascular risk: a report of the american college of cardiology/american heart association task force on practice guidelines. Circ.

[12] Jefferson K, Ahmed M, Choleva M, Mak S, Allard JP, Newton GE (2015). Effect of a sodium-restricted diet on intake of other nutrients in heart failure: implications for research and clinical practice. J Card Fail.

[13] Song EK, Kang SM (2017). Micronutrient deficiency independently predicts adverse health outcomes in patients with heart failure. J Cardiovasc Nurs.

[14] Campos CL, Wood A, Burke GL, Bahrami H, Bertoni AG (2019). Dietary approaches to stop hypertension diet concordance and incident heart failure: the multi-ethnic study of atherosclerosis. Am J Prev Med.

[15] Karanja N, Erlinger TP, Pao-Hwa L, Miller ER, Bray GA (2004). The DASH diet for high blood pressure: from clinical trial to dinner table. Cleve Clin J Med.

[16] Chiavaroli L, Viguiliouk E, Nishi SK, Blanco Mejia S, Rahelić D, Kahleová H (2019). Dash dietary pattern and cardiometabolic outcomes: an umbrella review of systematic reviews and meta-analyses. Nutrients.

[17] Mozaffarian D, Appel LJ, Van Horn L (2011). Components of a cardioprotective diet: new insights. Circulation.

[18] Mellen PB, Gao SK, Vitolins MZ, Goff DC (2008). Deteriorating dietary habits among adults with hypertension: DASH dietary accordance, NHANES 1988–1994 and 1999–2004. Arch Intern Med.

[19] Kim H, Andrade FCD (2016). Diagnostic status of hypertension on the adherence to the Dietary Approaches to Stop Hypertension (Dash) diet. Prev Med Rep.

[20] Drewnowski A (2010). The cost of US foods as related to their nutritive value. Am J Clin Nutr.

[21] Monsivais P, Rehm CD, Drewnowski A (2013). The DASH diet and diet costs among ethnic and racial groups in the United States. JAMA Intern Med.

[22] Liu Y, Eicher-Miller HA (2021). Food insecurity and cardiovascular disease risk. Curr Atheroscler Rep.

[23] Coleman-Jensen A, Rabbitt MP, Gregory CA, Singh A (2021). Household food security in the United States in 2020.

[24] Gundersen C, Hake M, Dewey A, Engelhard E (2020). Food Insecurity during COVID-19. Appl Econ Perspect Policy.

[25] Huizar MI, Arena R, Laddu DR (2021). The global food syndemic: The impact of food insecurity, Malnutrition and obesity on the healthspan amid the COVID-19 pandemic. Prog Cardiovasc Dis.

[26] Hanson KL, Connor LM (2014). Food insecurity and dietary quality in US adults and children: a systematic review. Am J Clin Nutr.

[27] Holben DH, Marshall MB (2017). Position of the academy of nutrition and dietetics: food insecurity in the united states. J Acad Nutr Diet.

[28] Ziliak JP, Gundersen C (2017). The health consequences of senior hunger in the United States: Evidence from the 1999–2010 NHANES.

[29] Berkowitz SA, Terranova J, Randall L, Cranston K, Waters DB, Hsu J (2019). Association between receipt of a medically tailored meal program and health care use. JAMA Intern Med.

[30] Berkowitz SA, Terranova J, Hill C, Ajayi T, Linsky T, Tishler LW (2018). Meal delivery programs reduce the use of costly health care in dually eligible medicare and medicaid beneficiaries. Health Aff (Millwood).

[31] Berkowitz SA, Delahanty LM, Terranova J, Steiner B, Ruazol MP, Singh R (2019). Medically tailored meal delivery for diabetes patients with food insecurity: a randomized cross-over trial. J Gen Intern Med.

[32] Glanz K, Rimer B, Viswanath K (2008). Health Behavior and Health Education: Theory, Research, and Practice.

[33] Heart Failure. Academy of Nutrition and Dietetics Evidence Analysis Library. https://www.andeal.org/topic.cfm?menu=5289. Published 2017. Accessed 2021

[34] American Heart Association. How much sodium should I eat per day. Rev ed. 2021. Available from: https://www.heart.org/en/healthy-living/healthy-eating/eat-smart/sodium/how-much-sodium-should-i-eat-per-day.

[35] American Diabetes Association. Find Your Balance When it Comes to Carbs. Rev ed. 2021. Available from: https://www.diabetes.org/healthy-living/recipes-nutrition/understanding-carbs.

[36] Girovich B. Hunger is Health: The Association Between Food Insecurity and Diabetes in the Primary Care Center (PCC) at Grady Hospital in Atlanta, GA. 2015. Unpublished Master’s of Public Health Thesis. Emory University, Rollins School of Public Health.

[37] Gundersen C, Engelhard EE, Crumbaugh AS, Seligman HK (2017). Brief assessment of food insecurity accurately identifies high-risk US adults. Public Health Nutr.

[38] Kaufman JS, Cooper RS (2001). Commentary: considerations for use of racial/ethnic classification in etiologic research. Am J Epidemiol.

[39] Williams DR, Lawrence JA, Davis BA (2019). Racism and health: evidence and needed research. Annu Rev Public Health.

[40] Calloway EE, Seligman HK, Boyd LW, Stern KL, Rosenmoss S, Yaroch AL (2019). Development and testing of the FRESH Foods Survey to assess food pantry clients’ dietary behaviours and correlates. Public Health Nutr.

[41] Lechner M (2010). The estimation of causal effects by difference-in-difference methods estimation of spatial panels. FNT Econometr.

[42] Williamson DA, Bray GA, Ryan DH (2015). Is 5% weight loss a satisfactory criterion to define clinically significant weight loss?. Obesity (Silver Spring).

[43] Savarese G, Lund LH (2017). Global public health burden of heart failure. Card Fail Rev.

[44] Berkowitz SA, Shahid NN, Terranova J, Steiner B, Ruazol MP, Singh R (2020). “I was able to eat what I am supposed to eat”– patient reflections on a medically-tailored meal intervention: a qualitative analysis. BMC Endocr Disord.

