# Supplementary material for: The impact of medically tailored meals and nutrition therapy on biometric and dietary outcomes among food-insecure patients with congestive heart failure: a matched cohort study
Source: BMC Nutr. 2022 Oct 3;8:108. doi: 10.1186/s40795-022-00602-y (PMC9528877; doi:10.1186/s40795-022-00602-y)
Supplement: Supplementary file 1 — Additional file 1: SupplementalTable 1. Demographics for individuals with pre- and post- weight and BMI data. [file 40795_2022_602_MOESM1_ESM.docx]

**Supplemental Table 1: Demographics for individuals with pre- and post- weight and BMI data**

|  | **Intervention Cohort**  **(N=26)** | **Matched-Control Cohort**  **(N=69)** | **Overall**  **(N=95)** |
| --- | --- | --- | --- |
| **Age** |  |  |  |
| Mean (SD) | 63.0 (13.4) | 62.8 (12.9) | 62.8 (13.0) |
| Median [Min, Max] | 63.0 [37.0, 91.0] | 63.0 [37.0, 92.0] | 63.0 [37.0, 92.0] |
| **Sex** |  |  |  |
| Female | 10 (38.5%) | 25 (36.2%) | 35 (36.8%) |
| Male | 16 (61.5%) | 44 (63.8%) | 60 (63.2%) |
| **Race and Ethnicity** |  |  |  |
| Black or African American, Non-Hispanic | 25 (96.2%) | 66 (95.7%) | 91 (95.8%) |
| White or Caucasian, Non-Hispanic | 1 (3.8%) | 2 (2.9%) | 3 (3.2%) |
| Hispanic | 0 (0%) | 1 (1.4%) | 1 (1.1%) |
| **Community Needs Index** |  |  |  |
| 4 | 10 (38.5%) | 27 (39.1%) | 37 (38.9%) |
| 5 | 16 (61.5%) | 41 (59.4%) | 57 (60.0%) |
| Missing | 0 (0%) | 1 (1.4%) | 1 (1.1%) |
| **Inpatient Admissions in the Previous Year** |  |  |  |
| Mean (SD) | 2.92 (2.34) | 3.22 (2.66) | 3.14 (2.57) |
| Median [Min, Max] | 2.00 [1.00, 8.00] | 3.00 [0, 9.00] | 3.00 [0, 9.00] |
| Missing | 2 (7.7%) | 0 (0%) | 2 (2.1%) |
