# Supplementary material for: The impact of medically tailored meals and nutrition therapy on biometric and dietary outcomes among food-insecure patients with congestive heart failure: a matched cohort study
Source: BMC Nutr. 2022 Oct 3;8:108. doi: 10.1186/s40795-022-00602-y (PMC9528877; doi:10.1186/s40795-022-00602-y)
Supplement: Supplementary file 2 — Additional file 2: SupplementalTable 2. Demographics for individuals with pre- and post- blood pressure data. [file 40795_2022_602_MOESM2_ESM.docx]

**Supplemental Table 2: Demographics for individuals with pre- and post- blood pressure data**

|  | **Intervention Cohort**  **(N=22)** | **Matched-Control Cohort**  **(N=49)** | **Overall**  **(N=71)** |
| --- | --- | --- | --- |
| **Age** |  |  |  |
| Mean (SD) | 63.4 (13.6) | 62.6 (14.0) | 62.8 (13.8) |
| Median [Min, Max] | 64.5 [37.0, 91.0] | 61.0 [38.0, 92.0] | 62.0 [37.0, 92.0] |
| **Sex** |  |  |  |
| Female | 8 (36.4%) | 21 (42.9%) | 29 (40.8%) |
| Male | 14 (63.6%) | 28 (57.1%) | 42 (59.2%) |
| **Race and Ethnicity** |  |  |  |
| Black or African American, Non-Hispanic | 21 (95.5%) | 48 (98.0%) | 69 (97.2%) |
| White or Caucasian, Non-Hispanic | 1 (4.5%) | 1 (2.0%) | 2 (2.8%) |
| **Community Needs Index** |  |  |  |
| 4 | 9 (40.9%) | 20 (40.8%) | 29 (40.8%) |
| 5 | 13 (59.1%) | 28 (57.1%) | 41 (57.7%) |
| Missing | 0 (0%) | 1 (2.0%) | 1 (1.4%) |
| **Inpatient Admissions in the Previous Year** |  |  |  |
| Mean (SD) | 2.85 (2.21) | 2.69 (2.31) | 2.74 (2.27) |
| Median [Min, Max] | 2.00 [1.00, 8.00] | 2.00 [0, 8.00] | 2.00 [0, 8.00] |
| Missing | 2 (9.1%) | 0 (0%) | 2 (2.8%) |
