# Supplementary material for: The impact of medically tailored meals and nutrition therapy on biometric and dietary outcomes among food-insecure patients with congestive heart failure: a matched cohort study
Source: BMC Nutr. 2022 Oct 3;8:108. doi: 10.1186/s40795-022-00602-y (PMC9528877; doi:10.1186/s40795-022-00602-y)
Supplement: Supplementary file 3 — Additional file 3: Supplemental Table 3. Pre-Post Changes in DietaryIntake of Food Groups, Measured inTimes Per Day (n=11). [file 40795_2022_602_MOESM3_ESM.docx]

| **Supplemental Table 3. Pre-Post Changes in Dietary Intake of *Food Groups*, Measured in Times Per Day (n=11)** | | | | | | | | | | | |
| --- | --- | --- | --- | --- | --- | --- | --- | --- | --- | --- | --- |
| **Food Group** | **Timepoint** | **Mean** | **Std Dev** | **Median** | **Min.** | **Max.** | **N** | **Difference** | **S-Statistic** | **P-Value** | **Cohen's d** |
| **Whole F & V^A^** | **Pre** | 2.05 | 1.81 | 1.16 | 0.29 | 4.87 | 11 | 0.95 | 10.00 | 0.334 | 0.5609 |
|  | **Post** | 3.00 | 1.56 | 3.29 | 0.58 | 5.58 |  |  |  |  |  |
| **Whole F & V, No Potatoes^B^** | **Pre** | 1.79 | 1.79 | 0.87 | 0.00 | 4.58 | 11 | 0.94 | 8.50 | 0.359 | 0.5648 |
|  | **Post** | 2.73 | 1.55 | 3.00 | 0.58 | 5.29 |  |  |  |  |  |
| **Whole Grain Foods^C^** | **Pre** | 0.70 | 0.87 | 0.58 | 0.00 | 3.00 | 11 | -0.39 | -9.50 | 0.219 | -0.5791 |
|  | **Post** | 0.31 | 0.39 | 0.29 | 0.00 | 1.00 |  |  |  |  |  |
| **Fast Foods^D^** | **Pre** | 0.47 | 0.30 | 0.58 | 0.00 | 0.87 | 11 | 0.03 | 1.50 | 0.961 | 0.0786 |
|  | **Post** | 0.50 | 0.37 | 0.58 | 0.00 | 1.16 |  |  |  |  |  |
| **High Sodium Foods^E^** | **Pre** | 1.00 | 0.79 | 0.71 | 0.00 | 2.29 | 11 | -0.48 | -11.00 | 0.148 | -0.7793 |
|  | **Post** | 0.52 | 0.35 | 0.58 | 0.00 | 1.00 |  |  |  |  |  |
| **High Saturated Fat Foods^F^** | **Pre** | 1.48 | 1.05 | 1.16 | 0.29 | 4.29 | 11 | -0.18 | -4.50 | 0.652 | -0.1640 |
|  | **Post** | 1.30 | 1.16 | 1.16 | 0.00 | 4.16 |  |  |  |  |  |
| **High Sugar Foods^G^** | **Pre** | 1.06 | 1.39 | 0.58 | 0.00 | 4.29 | 11 | 0.10 | -0.50 | 1.000 | 0.0702 |
|  | **Post** | 1.16 | 1.53 | 0.58 | 0.00 | 4.29 |  |  |  |  |  |
| A: Fruit + Salad + Fried Potatoes + Non-Fried Potatoes + Vegetables + Beans | | | | | | | | | | | |
| B: Fruit + Salad + Vegetables + Beans | | | | | | | | | | | |
| C: Whole Grain Bread + Cooked Whole Grains + Non-Sugary Cereal | | | | | | | | | | | |
| D: Fried Potatoes + Pizza + Tacos & Burritos + Hamburgers + Fried Chicken | | | | | | | | | | | |
| E: Heat-and-Serve + Processed Meat + Chips | | | | | | | | | | | |
| F: Fried Potatoes + Pizza + Tacos & Burritos + Heat-and-Serve + Hamburgers + Fried Chicken + Frozen Dessert + Cookies & Cakes + Chips | | | | | | | | | | | |
| G: Candy & Chocolates + Frozen Dessert + Cookies & Cakes + Sugary Cereals | | | | | | | | | | | |
